# Comparing the Efficacy of 5% EMLA Cream and 20% Benzocaine Gel on Gingival Tissue Surrounding Non-Carious Cervical Lesions for Reduction of Pain Following Rubber Dam Clamp Placement: A Split-Mouth Randomized Clinical Trial

**DOI:** 10.7759/cureus.63893

**Published:** 2024-07-05

**Authors:** Vidit B Naik, Deepak Sharma, Ashish K Jain, Rahul Rao, Amit Patil, Karan Akude, Ashima Jakhar

**Affiliations:** 1 Conservative Dentistry and Endodontics, Bharati Vidyapeeth (Deemed to be University) Dental College and Hospital, Navi Mumbai, IND; 2 Conservative Dentistry and Endodontics, Bharati Vidyapeeth (Deemed to be University) Dental College and Hospital, Navi Mumbai, IND

**Keywords:** topical anesthetics, benzocaine, emla, rubber dam, non-carious cervical lesions

## Abstract

Aim

The aim of this study was to evaluate the effectiveness of 5% eutectic mixture of local anesthetics (EMLA) cream and 20% benzocaine gel in reduction of pain during rubber dam clamp placement in the treatment of non-carious cervical lesions (NCCLs).

Methodology

In this split-mouth single-blind randomized clinical trial, 50 adult participants were selected from the outpatient department. The test group was treated using 5% EMLA cream for three minutes, and visual analog scale (VAS) scores were recorded. The comparison group was treated using 20% benzocaine gel and procedure was repeated as that in the test group. After recording the VAS scores, NCCLs in both the groups were restored using composite restoration.

Results

In the included 50 participants, 70% were males, with an age group of 31-67 years. The mean VAS score at 3 minutes in EMLA group was significantly lower than that in benzocaine group.

Conclusion

Application of 5% EMLA cream for 3 minutes showed greater pain reduction during rubber dam clamp placement as compared to 20% benzocaine gel in adult patients with NCCLs.

## Introduction

Three key benefits come from using a rubber dam during root canal therapy: protection, increased treatment effectiveness, and cross-infection control. Rubber dam protects the patient’s oropharynx from the possible aspiration or swallowing of instruments, medicaments, irrigating solutions, and tooth/material debris [1,2]. Non-carious cervical lesions (NCCLs) manifest themselves as a loss of mineralized tissue along the tooth surface near the gingival margin and typically extend from the cementoenamel junction onto the root surface [3]. It is known that because of the anatomy of NCCLs, which often present subgingival margins, there is need of gingival retraction, and retraction requires an effective gel containing a potent anesthetic salt [4].The gingival margins of teeth are exposed through the application of a metal clamp, also known as rubber dam clamp, which helps keep the rubber dam in place. This procedure may be painful especially in pediatric patients and often necessitates the use of local anesthetics for the patient’s comfort during the treatment.

Before applying the clamp, some dental professionals may administer papillary injections or administer no anesthetic at all [5]. EMLA cream is a 5% eutectic mixture of local anesthetics, manufactured by Astra Pharmaceuticals. It is a 1:1 oil/water emulsion of a eutectic mixture of 2.5% lidocaine and 2.5% prilocaine bases. EMLA cream has lower melting point, which allows it to become liquid in oral environment and aids in rapid transmucosal absorption to provide rapid onset anesthetic effect [6].

Previous literature has shown that 5% EMLA cream outperformed most of the other topical anesthetics, including 1% dyclonine, 10% benzocaine, 10% cocaine, 10% lidocaine, and placebo, in a research conducted by Roghani et al. [7] on an adult population. Vickers et al. [6] compared placebo to 5% EMLA cream, 5% xylocaine, and NUM (5% lignocaine, 1.7% amethocaine). It was discovered that all three topical medications could effectively lessen pain during needle insertion; however, EMLA was the most successful. A research conducted by Holst and Evers that involved adult subjects similarly found that EMLA is superior to "conventional" intraoral topical treatments in terms of palate effectiveness [8]. There are fewer studies in the literature comparing the efficacy of 5% EMLA cream and benzocaine gel on pain reduction following rubber dam clamp placement. The present split-mouth randomized clinical trial was conducted with a research question of whether which of the two topical anesthetics - 5% EMLA cream and benzocaine gel - is effective in reducing pain due to rubber dam clamp placement.

## Materials and methods

The present split-mouth randomized clinical trial was approved by the Institution Ethics Committee (IEC352072022) and was conducted according to guidelines proposed in Declaration of Helsinki. The article was written based on CONSORT statement [9] of randomized trials.

Participants

Study participants were selected from the OPD of the Department of Conservative Dentistry and Endodontics, Bharati Vidyapeeth (Deemed to be University) Dental College and Hospital, Navi Mumbai, Maharashtra, India. The participant inclusion criteria included participants who were older than 18 years of age irrespective of gender, those who had a bilateral NCCL in maxillary or mandibular premolars, and those who had healthy gingival tissue and were free from any pain or infection. Only participants who were willing to sign informed consent were selected for the study.

We excluded subjects with any systemic disease or allergies, presenting with pain or tooth mobility, or those who were unwilling to sign the consent forms.

Sample size

Sample size was based on the reported data for pain scores 3 minutes after EMLA application (3.00±2.038) and benzocaine application (4.00±2.124). Sample sizes of 50 in the test group (EMLA gel) and 50 in the control group (benzocaine gel) were required to achieve 90% power to detect a difference of 1.00 between the null hypothesis that both group means are 4.0 (control group benzocaine) and the alternative hypothesis that the mean of test group (EMLA) is 3.00 with known group standard deviations of 2.038 and 2.124 with a significance level (alpha) of 0.050 using a Paired sample t-test method. The significance level of the test was targeted at 0.0500. Thus, we planned to enroll a total of 50 samples for the study considering the dropouts.

Interventions

The participants were randomly divided into two groups: control group (n=50; 20% benzocaine gel used as a topical anesthetic agent) and test group (n=50; 5% EMLA cream used as a topical anesthetic agent).

Randomization, allocation concealment, and blinding

Simple randomization was done based on manually generated sealed envelopes containing application to the side of the jaw (left or right) and which topical anesthetics (EMLA or Benzocaine) to be used. After the subject was enrolled, the sealed envelope was opened only for that subject and treatment was assigned. Single blinding was carried out, i.e., the subjects were unaware whether he/she would receive which anesthetic gel on which side.

Clinical procedure

**Figure 1:**
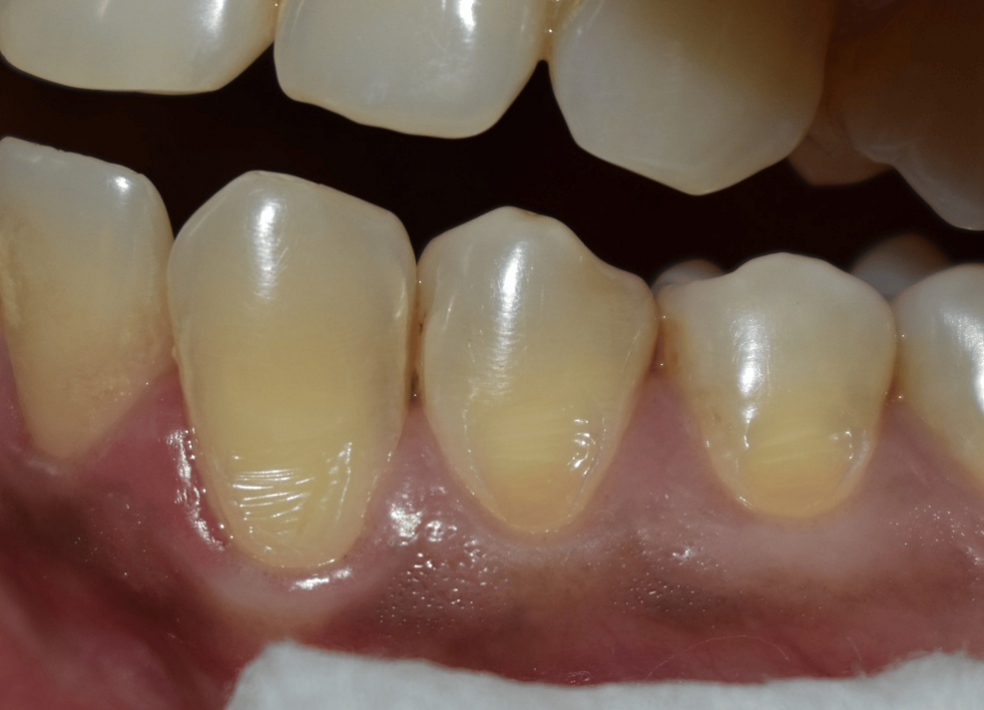
Tooth 34 with non-carious cervical lesion selected for the study.

Patients were instructed about all the steps of the treatment and the possible sensations they could experience during clamp adaptation. The tooth with NCCL was isolated with cotton rolls and saliva ejectors (Figure 1).

Control Group

In the control group, 20% benzocaine gel was placed extended approximately 3 to 5 mm below the crest of the gingival margin on both sides (facial and lingual) with the help of an applicator tip. After waiting for 3 minutes, the rubber dam was placed and Brinker's clamp #B6 was positioned on the tooth. The operator then immediately recorded the patient’s pain intensity and discomfort using visual analog scale (VAS) scores.

Comparison Group

**Figure 2:**
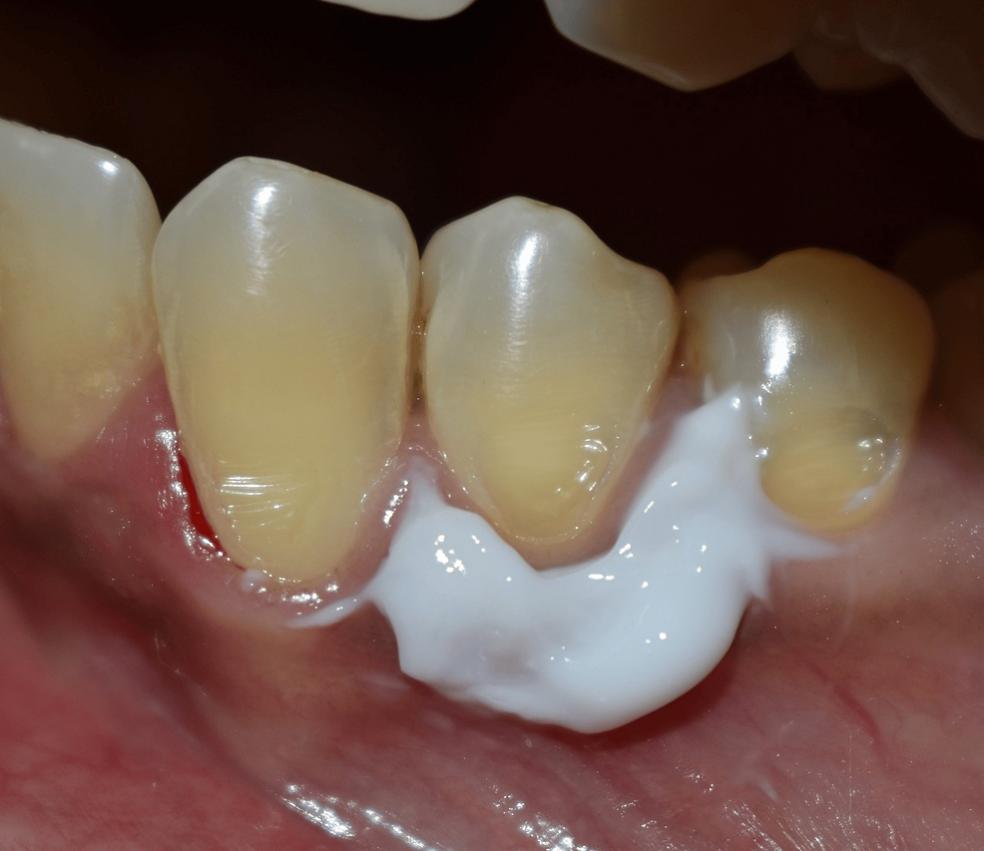
EMLA cream (5%) applied on gingival tissue surrounding tooth 34. EMLA, eutectic mixture of local anesthetics

**Figure 3:**
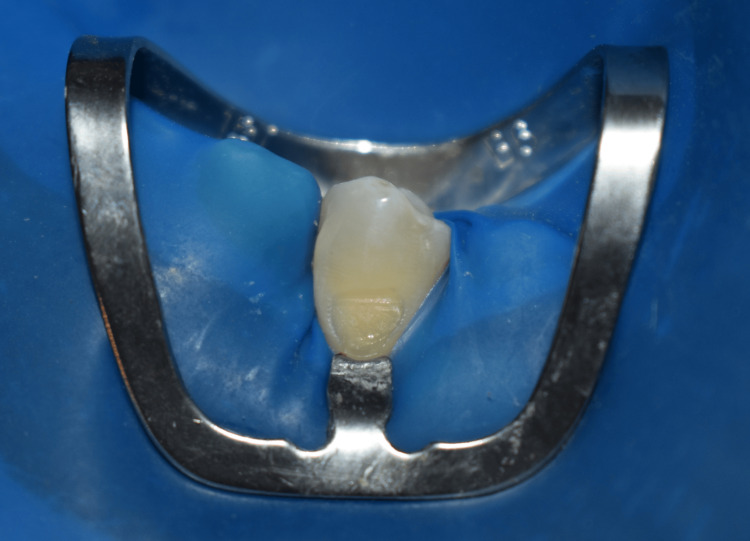
Brinker's clamp #B6 positioned on the tooth 34.

In the comparison group, 5% EMLA cream was placed extended approximately 3 to 5 mm below the crest of the gingival margin on both sides (facial and lingual) with the help of an applicator tip (Figure 2). After waiting for 3 minutes the rubber dam was placed and Brinker's clamp #B6 was positioned on the tooth (Figure 3). Similar to that of the control group, the operator recorded patient’s pain intensity and discomfort using VAS scores.

**Figure 4:**
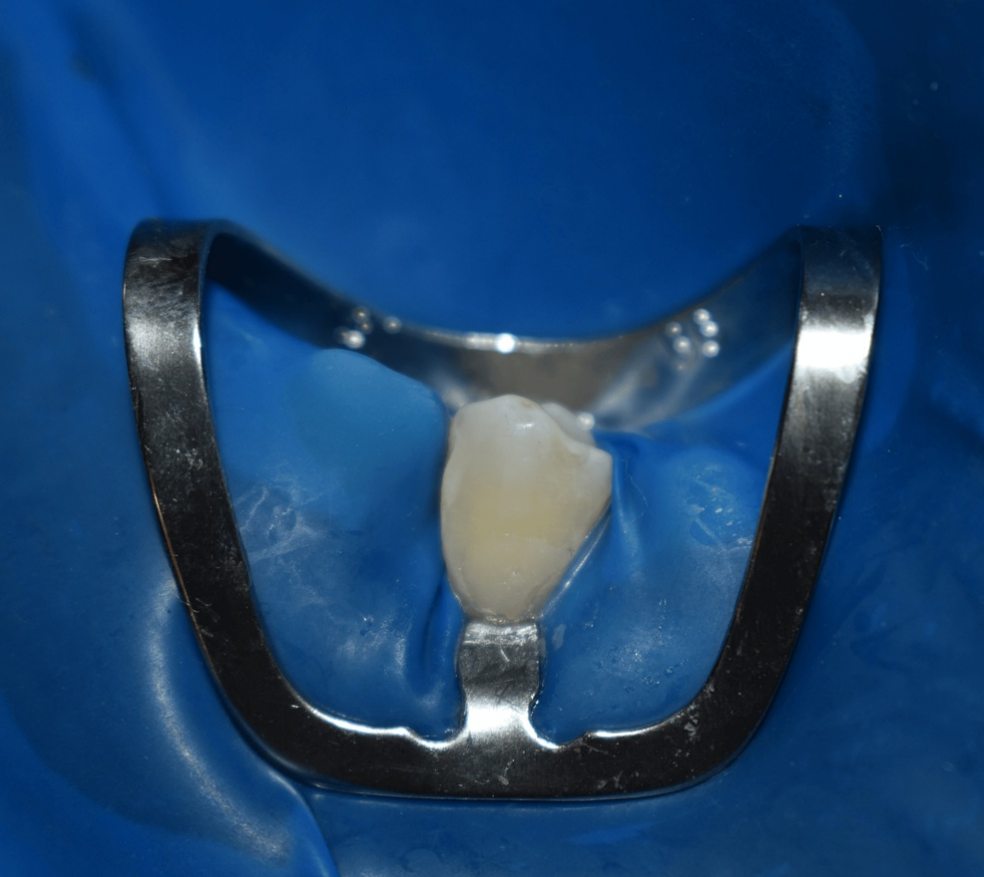
Restoration of the non-carious cervical lesion with appropriate adhesive protocol and composite resin.

Once adequate isolation was obtained, the restoration of the NCCL was carried out with composite resin and adhesive system in both groups (Figure 4).

Outcomes

A 100mm VAS scoring was used to measure the severity of pain interpreted as follows: 0, no pain; 1 to 3, mild pain; 4 to 6, moderate pain; 7 to 10, severe pain. If the subjects found the pain unbearable, a rescue anesthesia (2% lidocaine with epinephrine 1:100,000) was infiltrated in the region of concern. Pain scores were evaluated at 3 minutes’ duration.

Statistical analysis

The data obtained were compiled systematically into spreadsheets. The tables were titled appropriately and presented in Microsoft Excel (MS Excel 2016, Microsoft Corp., Redmond, WA). Statistical analysis was performed using MedCalc® Statistical Software Version 22.021 (MedCalc Software Ltd, Ostend, Belgium). For all the tests, p<0.05 was considered statistically significant at 95% confidence interval. Descriptive statistics were performed in terms of mean, standard deviation, frequency, and percentages. The two groups were compared for differences in pain using an unpaired t-test.

## Results

We included 50 participants in this study, with an age range of 18-67 years and a mean age of 51.68 (SD 9.03) years. Among the included participants, 35 were males, constituting the majority, while females comprised 15 participants. The demographic details of included participants are given in Table 1.

**Table 1:** Demographic characteristics of sample EMLA, eutectic mixture of local anesthetics

	Test group (5% EMLA)	Control group (20% benzocaine)
Number of participants (n)	50	50
Age of participants	51.68 ± 9.03	
Gender		
Males	35	35
Females	15	15

**Figure 5:**
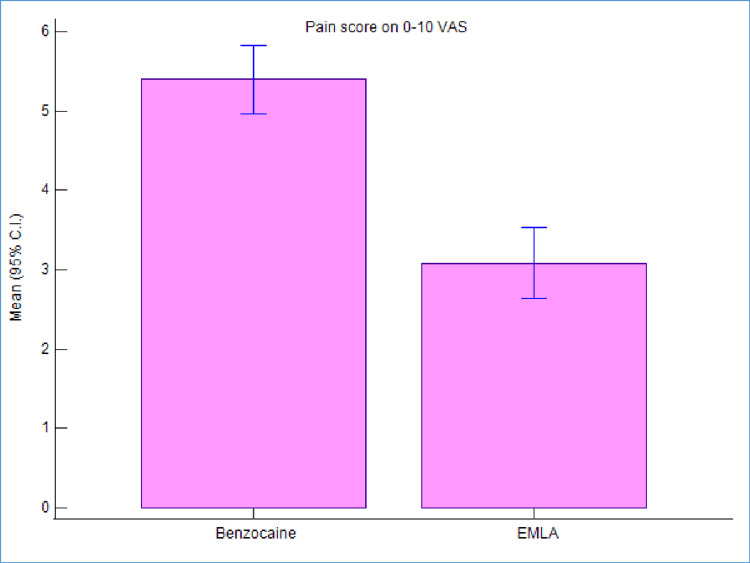
Mean VAS scores of benzocaine and EMLA at 3 minutes. EMLA, eutectic mixture of local anesthetics; VAS, visual analog scale

Based on the present split-mouth study design, each participant received two topical anesthetic agents (EMLA and benzocaine) on either side of their arch. The mean VAS score for the benzocaine group at 3 minutes was 5.40 and standard deviation (SD) was 1.51, while that for EMLA group at 3 minutes was 3.08 and SD was 1.57, as illustrated in Table 2 and Figure 5.

**Table 2:** Mean VAS scores and standard deviation for both the groups *The p-value was obtained using an unpaired t-test, highly significant EMLA, eutectic mixture of local anesthetics; VAS, visual analog scale

Study groups	N	Mean	Standard deviation	t-Value	p-Value
Control group (20% benzocaine)	50	5.40	1.51	-7.51	<0.001*
Test group 5% (EMLA)	50	3.08	1.57		

The mean scores of VAS scale at 3 minutes were statistically significant between the two groups(p<0.001). Mean score was significantly higher for the benzocaine group than the EMLA group. Thus, VAS scores at a duration of 3 minutes illustrated that 5% EMLA cream reduced pain significantly better than 20% benzocaine gel.

There was no significant difference in pain scores between males and females for either the benzocaine group or the EMLA group. There was no correlation between age and VAS scores in both groups.

## Discussion

Studies comparing the discomfort of restoring NCCLs with clamps are uncommon, despite the fact that this operation is frequently used [4]. According to previous research by Loguercio et al. [10], 70% of patients experienced pain while using a retraction cord or #212 clamp without first applying topical anesthetic agents. Most of the time in these circumstances, injectable anesthetic agent was needed. This study’s subjects reported less amount of pain and discomfort with EMLA cream than the control group.

EMLA cream may be specifically recommended in keratinized tissue than other topicals, despite significant drawbacks, such as a poor taste and time of administration. According to Holst and Evers [8], EMLA is more effective than traditional intraoral topical treatments in certain areas, such as the palate. Because of this, EMLA was chosen for this study's application around the gingiva prior to the implantation of dental dams.

The results of our study are in agreement with that reported by AlWeshah et al. [11], in which the authors compared 5% EMLA and 20% benzocaine as topical anesthetics for palatal injections. Authors concluded that EMLA was more effective than benzocaine. In a recent randomized controlled trial by Chugh et al. [12], comparison was done between EMLA topical application and injection, and it was concluded that compared to injection, EMLA delivered a good degree of anesthetic in the palatal tissue.

EMLA penetrates the oral mucosal membrane more deeply than other topical agents, roughly 5 mm as opposed to other topical solutions, which have shown around 2-3 mm depth of mucosal penetration, which is an advantage over conventional topical anesthetics. Unlike other topical anesthetics, which can only act on non-keratinized tissue, it can diffuse efficiently through keratinized tissue, such as the hard palate and gingival mucosa. Because it may more successfully permeate the buccal cortical plate to block A-delta, unmyelinated C-fibers, and nociceptive fibers, EMLA is thought to have bone effects [13].

In the present study, we used 100mm VAS scoring to evaluate the pain scores in subjects. The scoring criteria ranged from 0 to 10, with zero indicating no pain and 10 indicating severe pain [14]. According to Revill et al. [15], VAS lines shorter than 100mm tend to produce greater error variance. Hence, to avoid the error variance, 100mm VAS scoring was used in our study. Previous studies have reported certain complications with EMLA cream, such as allergies, anaphylaxis, burning sensation, and itching [16]. However, in our study, no adverse effects were observed among the participants.

The advantages of the present study include, firstly, the split-mouth design, which enabled each of the study subject to objectively compare pain with both materials. Secondly, strict randomization and concealment protocol was followed by the investigators, thus increasing the validity of this clinical trial. This study also showed certain limitations such as the reduced sample size, which may have affected the power of the study. Also, pain scores were recorded at only one time point, i.e., 3 minutes, which failed to provide details regarding the extent of anesthesia with EMLA and benzocaine. Future studies can be planned considering these limitations and comparing EMLA with other potent anesthetic solutions.

## Conclusions

The present study's limitation is the subjective nature of pain intensity and risk assessment, both of which are dependent on individual interpretation. Thus, within the limitations of this study, it can be concluded that application of 5% EMLA cream for 3 minutes showed greater pain reduction during rubber dam clamp placement as compared to 20% benzocaine gel in adult patients with NCCLs.

Meticulously designed clinical investigations are needed to further validate the utilization of newer forms of topical anesthetic agents for clamp adaptation in NCCLs with the objective of mitigating pain.
